# Supplementary material for: CryoWriter: a robotic solution for improved Cryo-EM grid preparation
Source: Nat Commun. 2026 May 30;17:7015. doi: 10.1038/s41467-026-73752-3 (PMC13392361; doi:10.1038/s41467-026-73752-3)
Supplement: Supplementary file 1 — Supplementary Information [file 41467_2026_73752_MOESM1_ESM.pdf]

Supplementary Tables

Method	Initial particle numbers	Final particle numbers	Retention ratio (or yield)	Concentration (mg/ml)	Resolution (Å)
Tobacco mosaic virus (TMV)					
Vitrobot	393,692	91,927[1]	23.34 %	10	2.30
cryoWriter	484,223	95,222	19.66 %	20	1.83
		25,681	5.30 %		2.08
Horse Spleen Apoferritin (apoF)					
Vitrobot	689,633	91,000[2]	13.19 %	1.1	2.34
cryoWriter	1,541,607	469,137	30.43 %	10	1.68
		127,449	8.26 %		1.89
cryoWriter	1,769,072	760,785	43.0%	5 (2 times writing)	1.71
Mouse heavy chain apoferritin (apoF)					
Leica EM GP2	1,104,665	411,705[3]	37.26 %	08	1.09
Transient receptor potential (TRP) melastatin 4 (TRPM4)					
Vitrobot	1,719,323	255,053[4]	14.83 %	01	2.80
cryoWriter	695,101	69,878	10.05 %	06	3.01
Streptavidin (SA)					
Vitrobot	1,346,980	45,686[5]	3.39 %	0.2	3.20
cryoWriter	190,686	47,564	24.94 %	03	2.97

Supplementary Table 1: Quantitative comparison of particle yield for final 3D reconstruction and concentration between cryoWriter and conventional plunge freezing.

**Sample**	**Apoferritin**			
Molecular weight	483 kDa			
Concentration [mg/mL]	5			
Writing type	Spiral			
Number of writings	2			
Spot-to-plunge time [s]	2	3	4	5
Micrographs	2	9	8	4
Pixel size [Å/px]	0.83			
Expected number of particles per µm^2 when assuming deposition of a 100nm layer of sample	1247 (2 x 623 due to double-writing)			
Observed number of particles per µm^2	2,674	3815	5,537	3,071

Supplementary Table 2: Particle density in cryo-EM grids prepared with different waiting times between writing and plunge-freezing. ApoF protein sample at a concentration of 5 mg/mL was written twice onto the same cryo-EM grid, followed by different waiting times before plunge-freezing. The images were recorded at strong defocus (i.e., high image contrast) on thin ice layers, so that all particles were strongly visible.

**Sample**	**Apoferritin**			
Molecular weight	483 kDa			
Concentration (mg/mL)	2	5	5	10
Writing type	Spiral	Spiral	Spiral	Spiral
Number of writings	1	1	2	1
Ice thickness assumption [nm]	100			
Micrographs	12	5	16,692	6,017
Pixel size [Å/px]	0.83	0.9	0.41	0.66
Spot-to-plunge time [s]	0.2	0.2	3	0.2
Expected number of particles per μm^2 when assuming deposition of a 100nm layer of sample	249	623	1,247	1,247
Observed number of particles per μm^2	25	250	1631	1505
Resolution	–	–	1.71	1.68
EMDB ID	–	–	EMD-55027	EMD-54957

Supplementary Table 3: Electron micrographs showing particle distribution with varying concentrations. The images were recorded at lower defocus (i.e., optimized for high resolution data collection, while providing lower contrast) on slightly thicker ice layers and in the vicinity of the carbon film. Only isolated particles suitable for high-resolution structural analysis were picked. This resulted in a lower particle count than in Supplementary Table 1.

Sample	TMV	Apoferritin		TRPM4	Strept-avidin
Microscope	TFS Titan Krios G4, CFEG, Falcon 4i				
Voltage [kV]	300				
Grid type	Quantifoil R 1.2/1.3 on Cu 300				Quantifoil Active
Energy Filter (10 eV)	Selectris X	–	–	–	Selectris X
Sample Conc. [mg/mL]	20	10	10	6	3
Writing type	Spiral	Spiral	Line	Spiral	Line
Capillary diameter [µm]	125	125	125	200	125
Nominal Magnification	270,000	120,000	96,000	165,000	165,000
Electron exposure [$e^-/Å^2$]	80	60	40	50	60
Defocus [µm]	0.35 to 0.85	0.5 to 2.0	0.5 to 2.0	0.35 to 0.85	1.0 to 2.0
Pixel size [Å]	0.46	0.66	0.83	0.732	0.732
Micrographs	5,824	6,121	4,923	3,535	2,209
Initial particle numbers	484,223	1,541,607	802,322	695,101	190,686
Extraction Box Size	512	448	320	440	256
Particles per micrograph	47	105	111	201	99
Final particle numbers	95,222	469,137	230,450	69,878	47,564
Symmetry imposed	Helical	O	O	C4	D2
Guinier Plot B-factor [$Å^2$]	18.7	51.7	65.8	66.66	105
FSC threshold	0.143				
Map resolution [Å]	1.83	1.68	2.02	3.01	2.97
EMPIAR ID	EMPIAR-13339	EMPIAR-13322	EMPIAR-13340	EMPIAR-13335	EMPIAR-13336
EMDB ID	EMD-55006	EMD-54957	EMD-54970	EMD-54984	EMD-55000
Fit PDB ID	6RLP	6PXM	6PXM	8RCR	6J6J

Supplementary Table 4: Statistical parameters of the determined protein structures from cryo-EM grids prepared with the cryoWriter.

Supplementary Figures

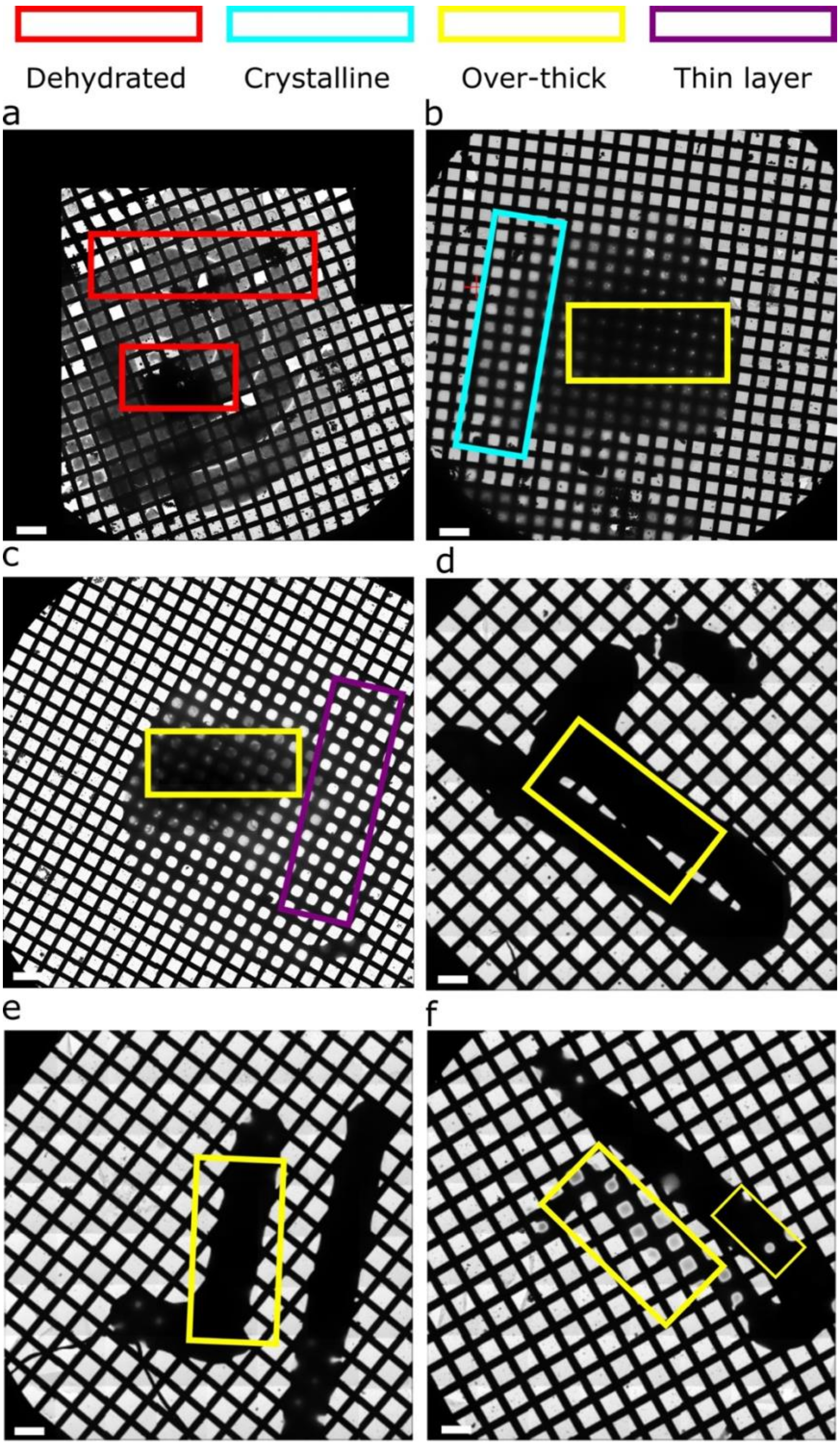

Supplementary Fig. 1 | Atlas images of grids, prepared with different writing patterns. a Overview image of the spiral writing pattern of the dehydrated apoF sample caused by evaporation before freezing. **b** Atlas image of overly thick ice at the center and crystalline ice. **c** Combination of both, overly thick and thin ice. **d-f** Line writing of the apoF sample leads to the formation of overly thick ice (scale bar = 200 µm).

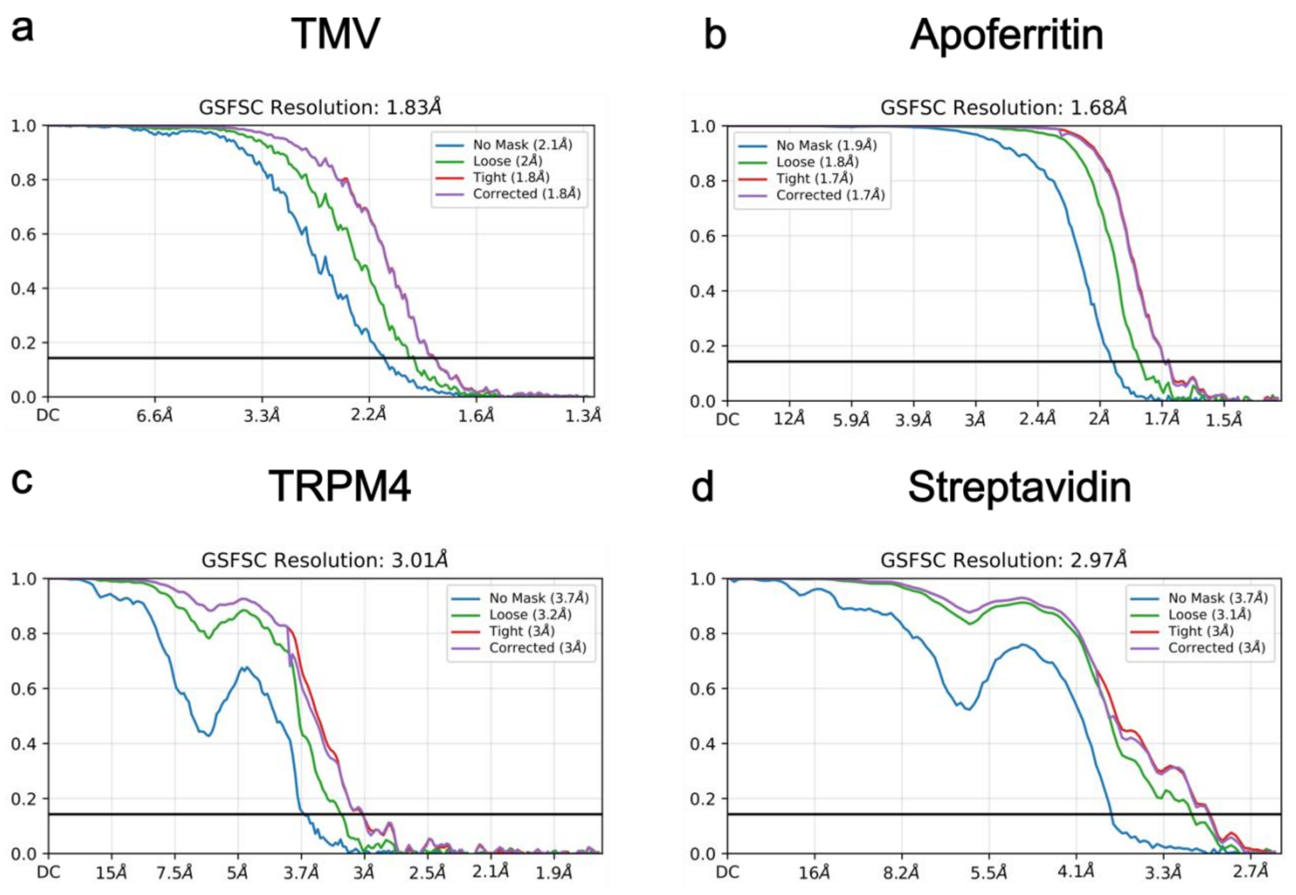

Supplementary Fig. 2 | Resolution analysis of cryo-EM reconstructions by Fourier Shell Correlation (FSC). a TMV at 1.83 Å resolution. **b** apoF at 1.68 Å resolution. **c** TRPM4 at 3.01 Å resolution. **d** Streptavidin and desthiobiotin complex at 2.97 Å resolution.

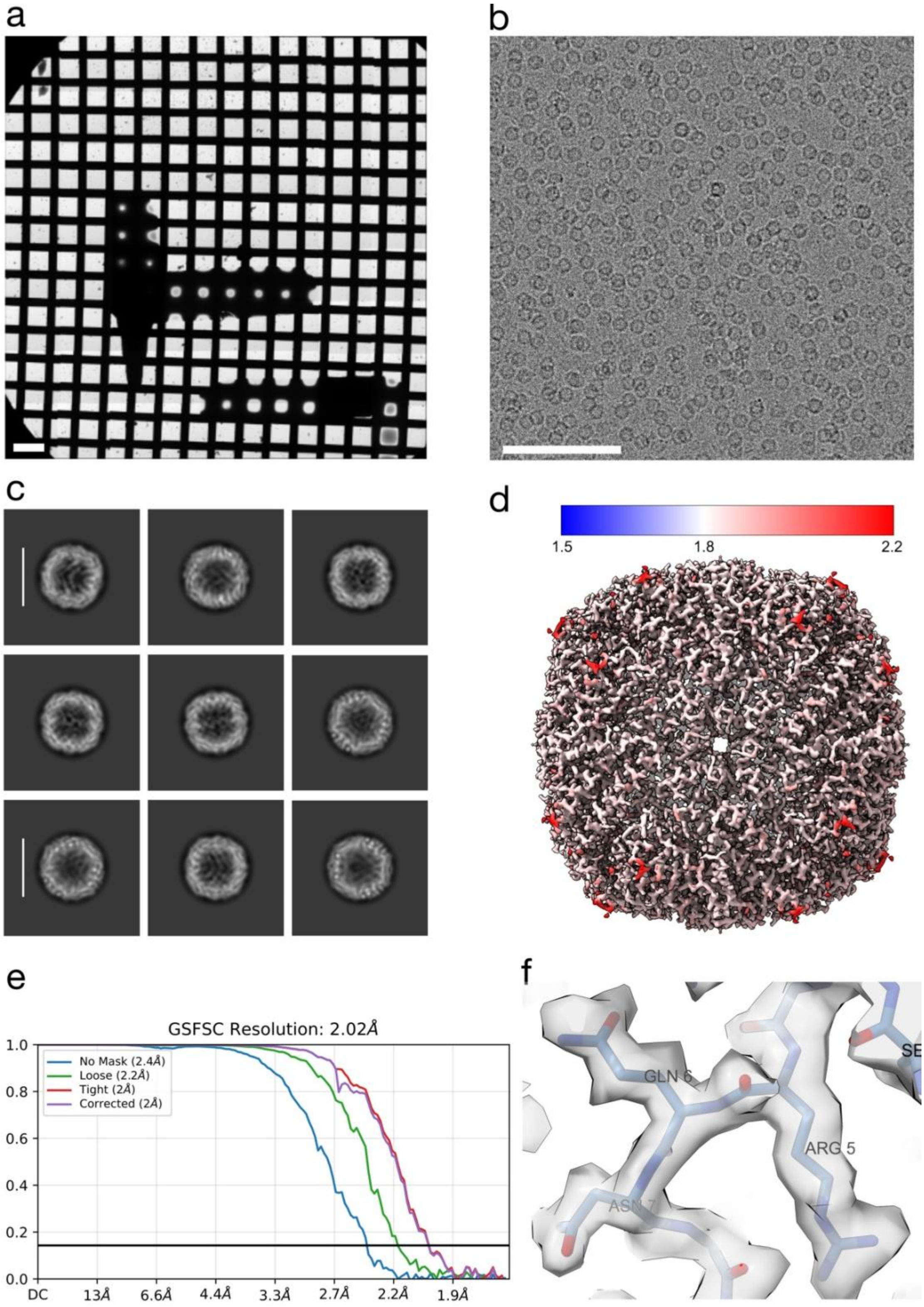

Supplementary Fig. 3 | High-resolution single particle reconstruction of apoF with line writing prepared using the cryoWriter. a Overview of the vitrified grid (scale bar = 200 µm). **b** Representative cryo-EM micrograph of apoF (Scale bar = 50 nm). **c** 2D class average of apoF (scale bar = 11 nm). **d** Local resolution map colored according to the assigned scale (scale in Å). **e** FSC curve showing the resolution at 2.02 Å. **f** An exemplary image showing the cryo-EM densities of apoF fit well into the atomic model.

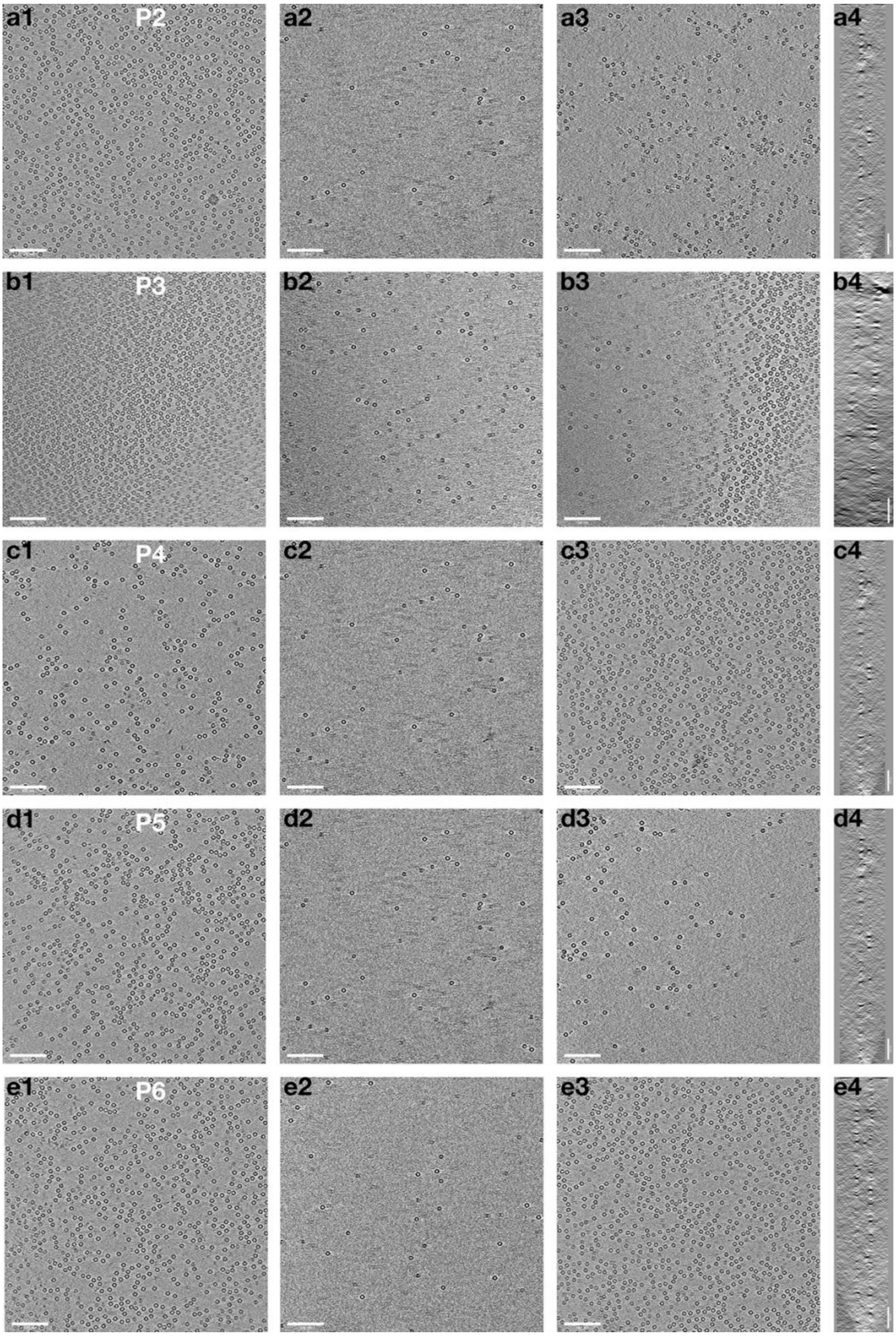

Supplementary Fig. 4 | Measurements of ice thickness and particle distribution in cryoWriter grids. a-e Panels from top to bottom are from positions 2 to 6 on the ApoF grid shown in Fig. 3a. Sub-panels 1 to 4 from left to right depict **1:** Tomographic reconstruction, showing the top layer of the 3D reconstruction. **2**: showing the central layer, and **3**: the bottom layer of the 3D reconstruction. **4**: The side-view of the 3D reconstruction allows to recognize the 3D distribution of particles to the two surface layers of the ice. (scale bars = 100 nm).

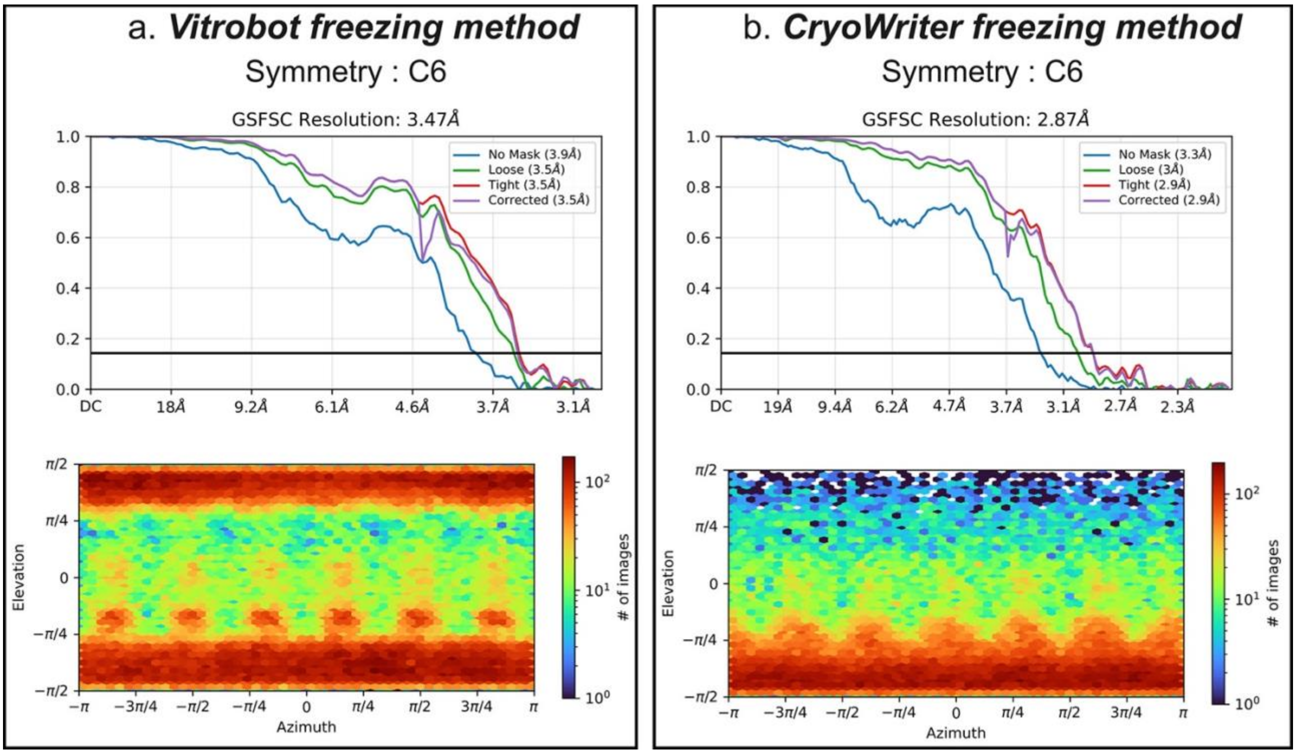

Supplementary Fig. 5 | Analysis of the NrS-1 particle orientation in cryo-EM grids. **a** Vitrobot freezing method with C6 symmetry applied, corresponding FSC curve at 3.47 A resolution, and a particle viewing direction distribution for 133,779 particles. The resolution improved when compared to C1 symmetry. **b** CryoWriter freezing method when applied to C6 symmetry, corresponding FSC curve showing 2.87 Å resolution and particle viewing direction distribution with 96,862 particles. The resolution improved when compared to C1 symmetry.

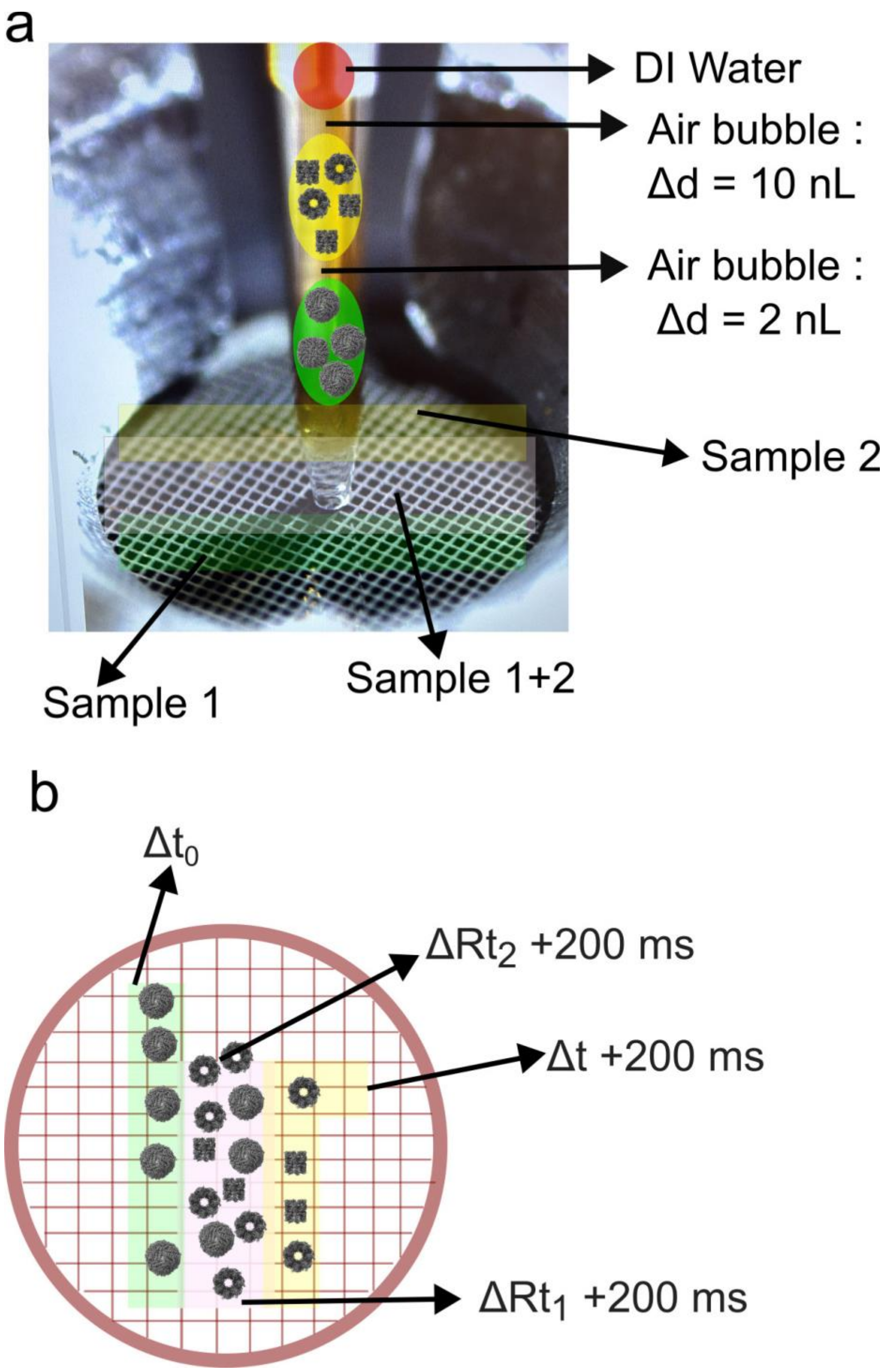

Supplementary Fig. 6 | Scheme of writing two different samples onto the same cryo-EM grid. **a** Schematic diagram illustrating these two different sample writing strategies on the same grid. **b** Schematic diagram illustrating time resolution approximation by writing two different samples on a cryo-EM grid.

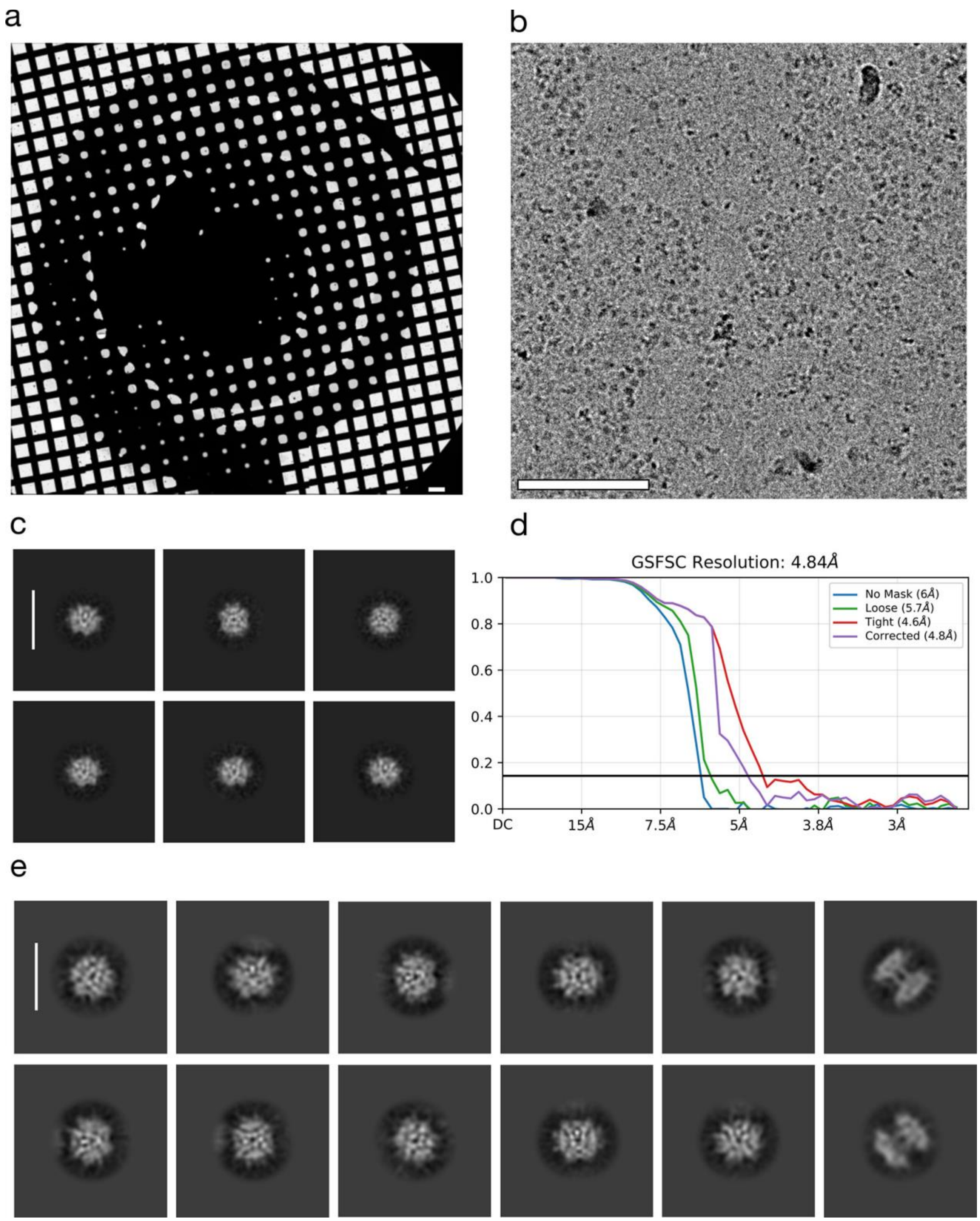

Supplementary Fig. 7 | Overview and 2D classification of preferential orientation of streptavidin protein. a Overview of the vitrified grid of streptavidin (scale bar = 200 µm). **b** Representative cryo-EM micrograph of the streptavidin protein alone (Scale bar = 50 nm). **c** 2D class average of streptavidin (scale bar = 9 nm). **d** FSC curve shows the resolution is at 4.84 Å due to strong preferential orientation. **e** 2D class averages of streptavidin showing preferential orientation frozen with Vitrobot (Scale bar = 7 nm).

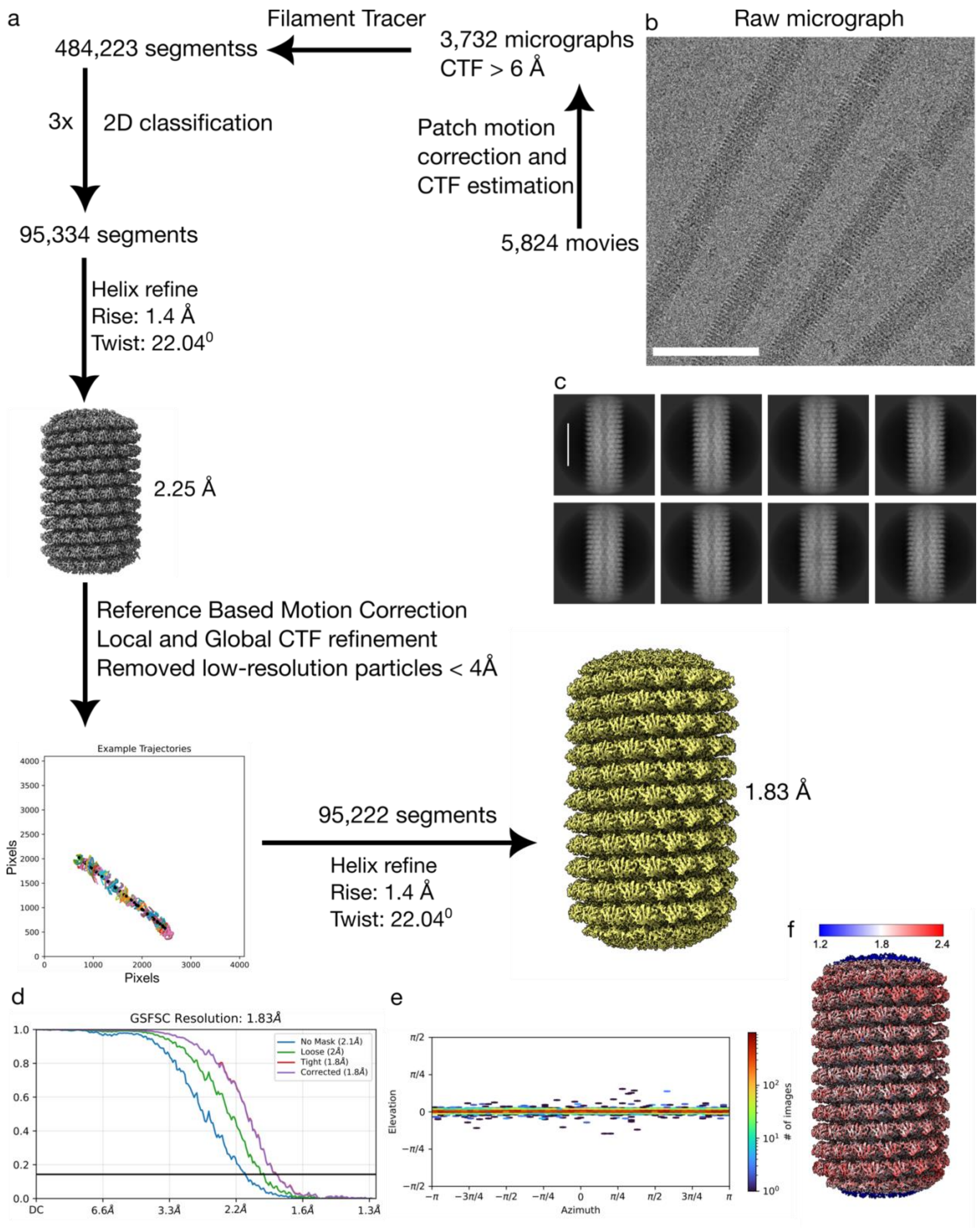

Supplementary Fig. 8 | Cryo-EM reconstruction of the TMV. a Schematic representation of cryo-EM data processing pipeline. **b** Representative motion corrected micrograph (scale bar= 50 nm). **c.** Selected 2D class averages (scale bar = 190 Å). **d** Gold standard Fourier shell correlation curve (GSFSC) at 0.143 cut-off was used to determine the overall resolution of the map. **e** Angular plot of the particles used for 3D reconstruction. **f** Local resolution map of the 3D reconstruction in front view. (Color bar in Å).

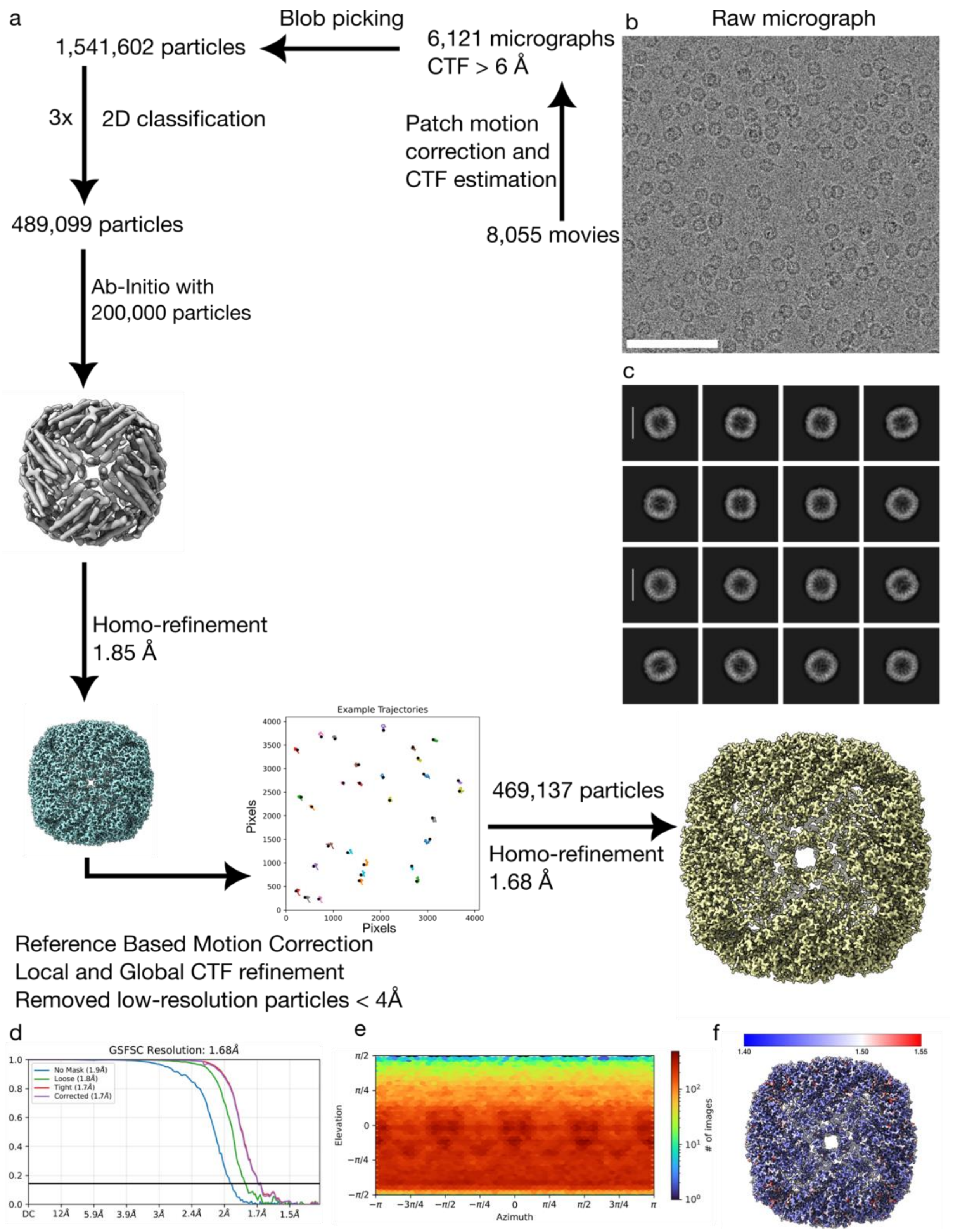

Supplementary Fig. 9 | Cryo-EM reconstruction of the apoF written in spiral pattern. **a** Schematic representation of cryo-EM data processing pipeline. **b** Representative motion corrected micrograph (scale bar= 50 nm). **c.** Selected 2D class averages (scale bar = 120 Å). **d** Gold standard Fourier shell correlation curve (GSFSC) at 0.143 cut-off was used to determine the overall resolution of the map. **e** Angular plot of the particles used for 3D reconstruction. **f** Local resolution map of the 3D reconstruction in front view. (Color bar in Å).

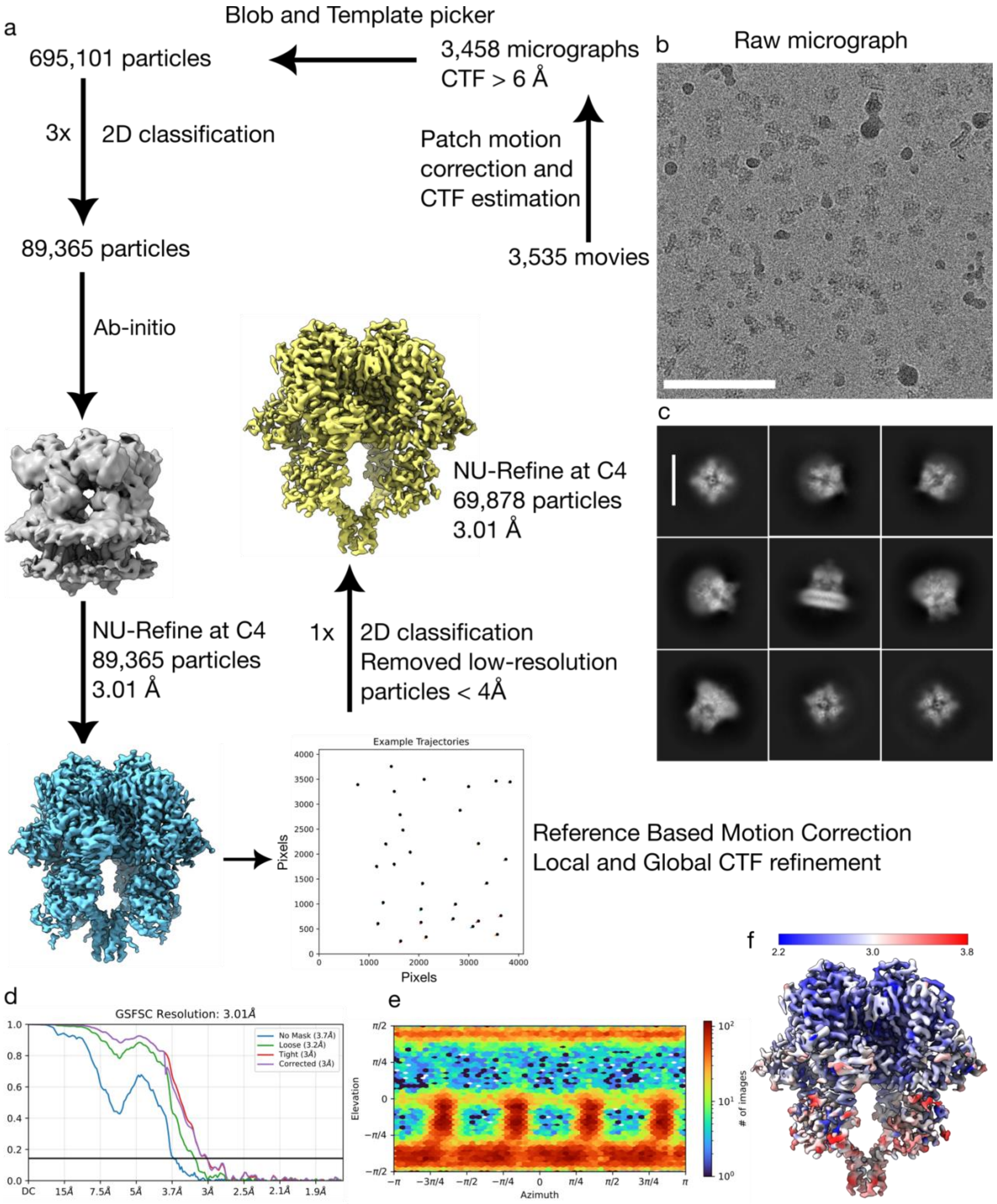

Supplementary Fig. 10 | Cryo-EM reconstruction of the TRPM4. a Schematic representation of cryo-EM data processing pipeline. **b** Representative motion corrected micrograph (scale bar= 50 nm). **c.** Selected 2D class averages (scale bar = 100 Å). **d** Gold standard Fourier shell correlation curve (GSFSC) at 0.143 cut-off was used to determine the overall resolution of the map. **e** Angular plot of the particles used for 3D reconstruction. **f** Local resolution map of the 3D reconstruction in front view. (color bar in Å).

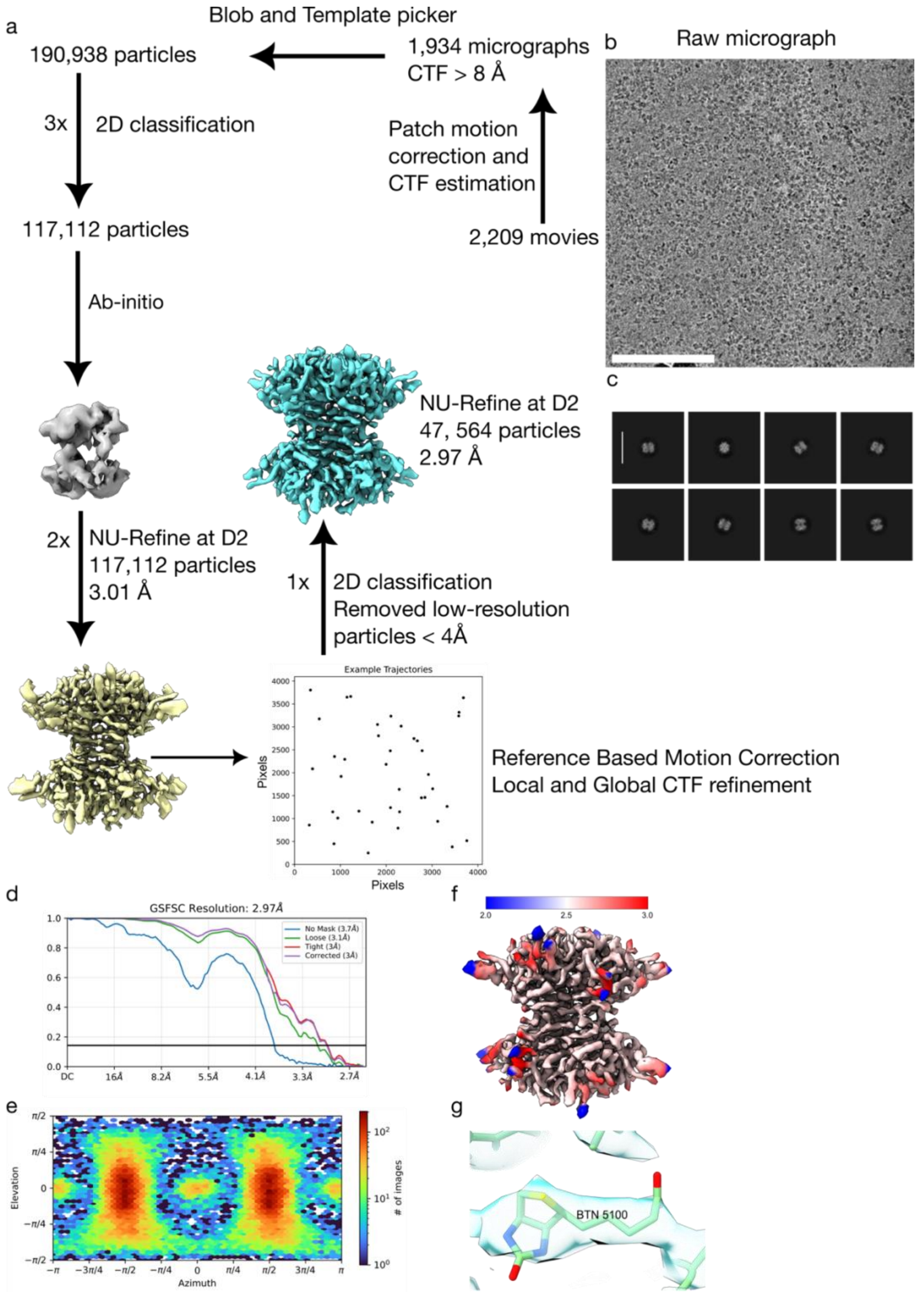

Supplementary Fig. 11 | Cryo-EM reconstruction of the Strepatividin-biotin complex. **a** Schematic representation of cryo-EM data processing pipeline. **b** Representative motion corrected micrograph (scale bar = 50 nm). **c.** Selected 2D class averages (scale bar = 140 Å). **d** Gold standard Fourier shell correlation curve (GSFSC) at 0.143 cut-off was used to determine the overall resolution of the map. **e** Angular plot of the particles used for 3D reconstruction. **f** Local resolution map of the 3D reconstruction in front view. (color bar in Å). **g** biotin (ligand) density bound to Strepatividin in the on-grid mixing writing.

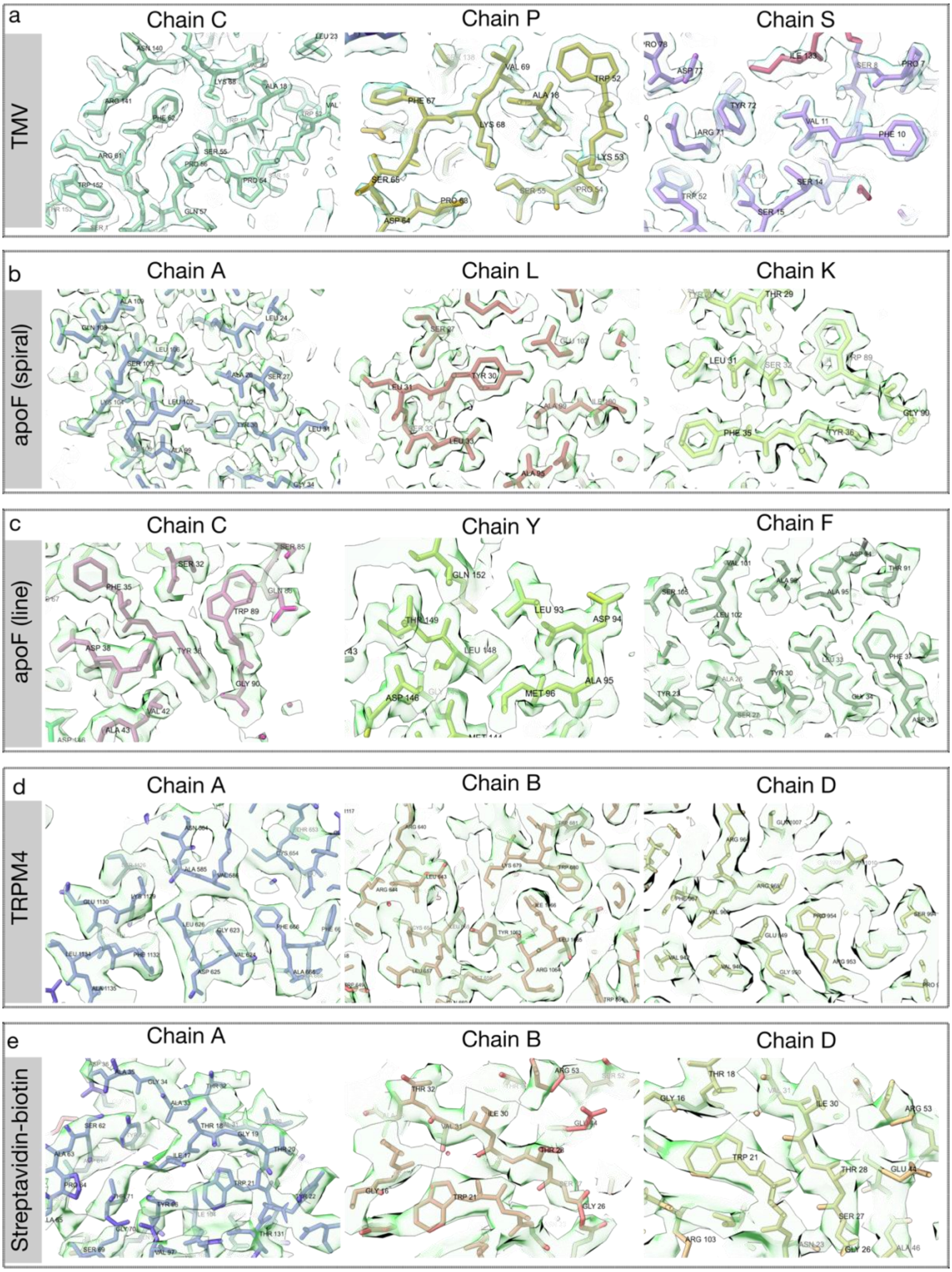

Supplementary Fig. 12 | Exemplary cryo-EM density maps fitted with model. **a** cryo-EM densities for the different chains for TMV map. **b** cryo-EM densities for the different chains for apoF map written in spiral pattern. **c** cryo-EM densities for the different chains for apoF map written in line pattern. **d** cryo-EM densities for the different chains for TRPM4 map. **e** cryo-EM densities for the different chains for Streptavidin-biotin map.

Supplementary Texts

Supplementary Text 1: CryoWriter freezing workflow

1. Start by filling the cryopot with liquid nitrogen, followed by filling the ethane pot with liquification ethane, and maintaining the ethane temperature between 180 and 183 °C.
2. The Eppendorf tube containing the sample is placed in a nano-incubator, with the temperature adjusted according to experimental requirements. In routine operation, the nano-incubator is maintained between 4 °C and 8 °C.
3. The relative humidity control is then activated, and dew point offset temperatures are set for the tweezer, capillary, and launchpad. These values are user-defined; typical operating conditions are 60-70% relative humidity and a dew point temperature offset of +2 °C.
4. Grids are subsequently loaded into the grid loading station. Using the gripper/tweezer, each grid is automatically transferred to the glow discharge station to render the surface hydrophilic. Glow discharge parameters, such as duration and discharge current, are user-specified. Alternatively, grids may be pre-glow-discharged outside the cryoWriter system before loading.
5. Following glow discharge, the tweezer automatically transfers the grid to the launchpad, which is maintained at the dew point temperature, typically 16-18 °C.
6. The capillary is then positioned in the nano-incubator to aspirate the desired volume of sample, with both volume and aspiration speed determined by the user. The capillary is subsequently moved back to the launchpad, where the grid is positioned for sample deposition.
7. Sample application parameters, including writing pattern (e.g., spiral or linear), writing speed, deposition rate, and spacing between writing are user controlled.
8. Finally, the grid is plunge-frozen into liquid ethane, after which it is automatically transferred into a storage puck.

Supplementary Text 2: Calculation of the expected particle numbers in an image

Calculation of protein particles per field of view in an electron microscope

Apoferritin is a spherical particle with 24-fold symmetry. The particles have a molecular weight w of 483 kDa or 483,000 g/mol, and we assume a mass concentration c of 10 mg/mL or 10 g/L. The mass concentration can be expressed as particle concentration with:

$$C = \frac{c \times NA}{w} \tag{1}$$

with $NA = 6.022 \times 10^{23}$ particles/mol being Avogadro's number. The apoferritin sample therefore was available at a concentration of

$$C = \frac{10\ g/L\ \times\ 6.022 \times 10^{23} particles/mol}{483 \times 10^{3}\ g/mol} = 1.247\ \times 10^{19} particles/L \tag{2}$$

or $1\ pL\ =\ 12.47\ \times\ 10^{6}\ particles$

Let us assume an ice thickness of 100 nm. The sample volume within a square micrometer is then 0.1 μm^3. Using the fact that 1 μm^3 = 1 x 10^{-3} pL, we note that in a square micrometer we should encounter a sample volume of 1 x 10^{-4} pL. That volume in the above example would contain 1247 particles, or result in a particle density of 1247 particles per square micrometer.

Supplementary Text 3: Calculation of expected trace thickness

Variable definition:

ω	= angular velocity [rad/s]
θ	= azimuth angle of of the spiral [rad]
t	= time [s]
r	= radius of the spiral [m]
a_c	= acceleration of the capillary during writing [m/s^2]
v	= speed of the capillary on the sample [m/s]
Q	= flow rate of the sample [L/s]
V	= dispensed sample amount [L] = $A \times h$
A	= surface area covered by sample [m^2]
h	= initial height of the written sample trace on the grid [m]
d	= initial diameter of the written sample trace on the grid [m]

Capillary writing at constant angular velocity and constant dispensing rate

When writing the sample onto the grid in a spiral pattern at a constant angular velocity $(\omega = const.)$, leading to a constantly increasing azimuth angle ($\theta(t) = \omega \times t$, with t being time), and using a slowly increasing radius $(r(t))$, a curved path is formed during which the capillary is moving at increasing speed when writing from the center to the outside of the spiral:

$$v(t) = \omega \times r(t) \quad (1)$$

The capillary is exposed to an acceleration $a_c(t)$ as:

$$a_c(t) = \frac{v(t)}{t} = \frac{\omega \times r}{t} \quad (2)$$

Assuming a constant width d of the written trace on the grid, the covered area on the grid per time is:

$$A(t) = v(t) \times t \quad (3)$$

If the sample is dispensed at a constant rate (Q), e.g., $Q = 1\ nL/s$, then the amount (V) of dispensed sample after a certain time is $V(t) = Q \times t$. The height before any evaporation of the written trace is $h(t) = \Delta V(t)/\Delta A(t)$.

The acceleration of the writing capillary a_c then leads to a decrease of the initial trace height h as:

$$h(t) = \frac{\Delta V}{\Delta A(t)} = \frac{\Delta V}{v \times t} \quad (4)$$

This shows that writing at constant angular velocity and constant dispensing rate leads to the generation of a spiral pattern that starts in the center with slower movements and finishes towards the outside with faster capillary movements, resulting in a decreasing sample thickness towards the outer areas of the spiral. This effect can be partly compensated with controlled drying, which would affect the first-written center of the spiral more than the outer areas of the spiral.

References

1. Song, B. *et al.* Capabilities of the Falcon III detector for single-particle structure determination. *Ultramicroscopy* **203**, 145–154 (2019).
2. Koning, R. I. *et al.* Automated vitrification of cryo-EM samples with controllable sample thickness using suction and real-time optical inspection. *Nat. Commun.* **13**, 2985 (2022).
3. Küçükoğlu, B. *et al.* Low-dose cryo-electron ptychography of proteins at sub-nanometer resolution. *Nat. Commun.* **15**, 8062 (2024).
4. Ekundayo, B. *et al.* Identification of a binding site for small molecule inhibitors targeting human TRPM4. *Nat. Commun.* **16**, 833 (2025).
5. Fan, X. *et al.* Single particle cryo-EM reconstruction of 52 kDa streptavidin at 3.2 Angstrom resolution. *Nat. Commun.* **10**, 2386 (2019).
