# Supplementary material for: CryoWriter: a robotic solution for improved Cryo-EM grid preparation
Source: Nat Commun. 2026 May 30;17:7015. doi: 10.1038/s41467-026-73752-3 (PMC13392361; doi:10.1038/s41467-026-73752-3)
Supplement: Supplementary file 2 — Description of Additional Supplementary Files [file 41467_2026_73752_MOESM2_ESM.pdf]

Description of Additional Supplementary Files

File name: Supplementary Movie 1
Description: The cryoWriter in operation.

File name: Supplementary Movie 2
Description: Spiral writing.

File name: Supplementary Movie 3
Description: Demonstration of the writing of two separate samples Nrs-1 and apoF onto the same Grid.

File name: Supplementary Movie 4
Description: Demonstration of the writing of two separate samples streptavidin and biotin onto the same grid.
