# Supplementary material for: CryoWriter: a robotic solution for improved Cryo-EM grid preparation
Source: Nat Commun. 2026 May 30;17:7015. doi: 10.1038/s41467-026-73752-3 (PMC13392361; doi:10.1038/s41467-026-73752-3)
Supplement: Supplementary file 8 — Transparent Peer Review file [file 41467_2026_73752_MOESM8_ESM.pdf]

nature portfolio

Peer Review File

CryoWriter: A Robotic Solution for Improved Cryo-EM Grid Preparation

Corresponding Author: Professor Henning Stahlberg

This file contains all reviewer reports in order by version, followed by all author rebuttals in order by version.

Version 0:

Reviewer comments:

Reviewer #1

(Remarks to the Author)
The manuscript by Chinmaya et al., discusses recent developments for the cryowriter system, an alternative approach to sample preparation in cryoEM. This is a quickly moving field and one that would benefit from some of the additional observations made within the paper. There are some interesting points raised around sample dilution and degradation and this builds on some of the previous observations seen for other systems such as Chameleon. At times it would benefit from additional descriptions of the data. I had some suggestions and comments that the authors may wish to address;

When discussing the “high resolution cryo-EM from cryowriter grids” the authors state how they changed certain parameters to get reproducible grids. However, little detail is given for those in the field also developing similar systems and approaches it would be good this to be expanded to discuss how much these factors were changed if any dominate more than others.

A small point but should it not be high resolution cryo-EM data from cryowriter grids, rather than just high resolution cryoEM?

The next section discusses the concentration needed and states how they are slightly higher than for blotting approaches. It would be good here to state the typical concentration used for structures solved by a blotting approach.

Page 7, paragraph 3, second line discusses the air water interface and previous studies but give no references. These should be added.

When discussing the NrS-1 DNA polymerase how have others overcome the preferred orientation problem given it is stated as a widely studied protein by cryo-EM. Is this through brute force, detergents, stage tilts? It would be interesting to see how others have done this. What sort of resolutions have they achieved?

The ability to mix two different lines of protein is interesting, I wondered if the authors had considered doing this not just to trigger reactions but to change pH etc… it could be very interesting to generate a pH range across the grid and see how the protein responds.

I was interested in how the particles themselves maybe of better quality by this approach. There is some evidence (mainly discussed at conferences) that by using these different approaches the quality of the data itself can be improved. This is hard to quantify but trends can be seen with lees particles removed for grids made on the chameleon for example. I noticed for TMV that a lot of particles went into the final reconstruction. For the other datasets after the initial 2D run where poorly picked particles and “junk” are removed what is the % of particles that make it through to the end and how does this compare to datasets published using the vitrobot for example?

No conflict of interest is stated but are any of the authors are also involved with the commercialisation of the cryoWriter and would this form a conflict?

Minor edits
There are some cases of one or two sentence paragraphs such as the third paragraph of the results it feels like these could possibly be incorporated in to other paragraphs.
H. sapiens and E. coli should be in italics.

Occasionally it states cryoEM rather than cryo-EM
Figure S7, for the FSCwhy does this extend to 1.3A, it would be more appropriate to extend to ~3A.

Reviewer #2

(Remarks to the Author)
The paper “CryoWriter: A Robotic Solution for Improved Cryo-EM Grid Preparation” by K.V. Chinmaya describes their experience of using a new instrument for cryo-EM grid preparation and its potential application for time-resolved cryo-EM studies. The authors characterized how the parameters of the sample preparation technique affect the properties of the amorphous ice and also demonstrated high-resolution structures of several biomolecules. The work provides a well-structured study that will be interesting for a broad audience as a single instruments provides an opportunity to both prepare high quality samples and perform time-resolved studies. However, it would be more convincing if several intermediates at different times were shown to demonstrate real time-resolved possibility.

Comments:

1. When the authors apply 2 samples at the same grid and wait 200 ms before freezing, did they observe a set of intermediate structures that correspond to different times after the interaction?
2. In the movie that demonstrated the U-shaped movement of the capillary, the liquid with the first sample distributes quite a lot, and when the capillary distributes the second sample, it is seen that it mixes with the first sample. Why didn’t the authors observe a mixture of both biomolecules everywhere?
3. The authors showed that the ice thickness depends on many parameters, such as the capillary movement speed; time after applying the sample, temperatures, and so on. Did the authors consider selecting the parameters such that they would have a uniformly thin amorphous ice layer, which could be beneficial for imaging small molecules?

Minor comments:
1. Sometimes the same information is repeated in neighboring paragraphs, such as about the temperature of the system (pages 4 and 5) and about the increasing concentration of salts that is causing denaturation of the samples (pages 6 and 7).
2. How accurately can the authors control the temperature of all the components of the system? If they were keeping the temperature of the grid 0.1 degree above the dew point, they could probably significantly reduce the degree of evaporation.
3. Did the authors estimate the degree of evaporation?
4. It could be useful to indicate the actual temperature of the liquid ethane thermostat instead of indicating that it is slightly above -183C.
5. The authors compare their instrument with different instruments for automatic cryo-EM grid preparation. However, it would probably be useful for the readers to include several sentences about existing methods for time-resolved cryo-EM to understand where the proposed technique stands (for example, a review of different methods is given in doi.org/10.1016/j.str.2022.11.014).

Version 1:

Reviewer comments:

Reviewer #1

(Remarks to the Author)
I feel this will make for a valuable contribution to the field and the amendments made have improved the clarity and I feel it is suitable for publication.

Reviewer #2

(Remarks to the Author)
The authors of the paper “CryoWriter: A Robotic Solution for Improved Cryo-EM Grid Preparation” have significantly improved their manuscript and addressed most of the questions, however, a single questions remained:

1. “The SI Video4 demonstrates the overlay of the atlas from the electron microscope and the video writing of two samples for NrS-1 and apoF mixing.” Unfortunately, The SI Video4 is not accessible in the “Manuscript Items” (please see the screenshot below):

Open Access This Peer Review File is licensed under a Creative Commons Attribution 4.0 International License, which permits use, sharing, adaptation, distribution and reproduction in any medium or format, as long as you give appropriate credit to the original author(s) and the source, provide a link to the Creative Commons license, and indicate if changes were made.
In cases where reviewers are anonymous, credit should be given to 'Anonymous Referee' and the source.
The images or other third party material in this Peer Review File are included in the article's Creative Commons license, unless indicated otherwise in a credit line to the material. If material is not included in the article's Creative Commons license and your intended use is not permitted by statutory regulation or exceeds the permitted use, you will need to obtain permission directly from the copyright holder.
To view a copy of this license, visit https://creativecommons.org/licenses/by/4.0/

REVIEWER COMMENTS

Reviewer #1 (Remarks to the Author):

The manuscript by Chinmaya et al., discusses recent developments for the cryowriter system, an alternative approach to sample preparation in cryoEM. This is a quickly moving field and one that would benefit from some of the additional observations made within the paper.
There are some interesting points raised around sample dilution and degradation and this builds on some of the previous observations seen for other systems such as Chameleon. At times it would benefit from additional descriptions of the data. I had some suggestions and comments that the authors may wish to address;

We thank the reviewer for the positive comments.

When discussing the "high resolution cryo-EM from cryowriter grids" the authors state how they changed certain parameters to get reproducible grids. However, little detail is given for those in the field also developing similar systems and approaches it would be good this to be expanded to discuss how much these factors were changed if any dominate more than others.

We thank the reviewer for the positive comments. Now we have discussed in line numbers 139 to 148: " During the initial optimization, constant writing start and end speeds (2 mm/s and 8 mm/s, respectively) were used, which resulted in a mixture of thick and thin ice. Modulating the writing speed enabled the generation of a controlled gradient of amorphous ice. The capillary–grid distance was maintained at a constant value of 3 µm throughout the writing process to ensure uniform sample distribution over the defined surface area of the grid. The chamber humidity was maintained at 60–70% to prevent sample drying. When the humidity dropped below 60%, rapid evaporation of the thin liquid film was observed, leading to dehydration and formation of crystalline ice (**Suppl. Fig. S1**). At slower writing speeds (2 mm/s), dispensing larger volumes of sample (5–8 nL) frequently resulted in thicker ice, particularly during line writing."

In the cryoWriter system, to get reproducible ice, the settings for writing speed, writing area and dispense volume play a major role. We have highlighted the writing parameters for different biomolecules in the results section.

A small point but should it not be high resolution cryo-EM data from cryowriter grids, rather than just high resolution cryoEM?

We agree, we have changed it in the manuscript, highlighted in yellow colour in line 146

The next section discusses the concentration needed and states how they are slightly higher than for blotting approaches. It would be good here to state the typical concentration used for structures solved by a blotting approach.

We thank the reviewer for the comments. We have now included a concentration comparison between the blotting approach in the supplement. Table 1 is highlighted in the manuscript in the line numbers 178 - 180.

Page 7, paragraph 3, second line discusses the air water interface and previous studies but give no references. These should be added.

We thank the reviewers for pointing this out. We have now added the reference in line 226.

When discussing the NrS-1 DNA polymerase how have others overcome the preferred orientation problem given it is stated as a widely studied protein by cryo-EM. Is this through brute force, detergents, stage tilts? It would be interesting to see how others have done this. What sort of resolutions have they achieved?

We clarified the statement that other DNA-binding proteins are widely studied by Cryo-EM. NrS-1, which others in our group investigate, was found to be a challenging target due to strong orientational bias.

The ability to mix two different lines of protein is interesting, I wondered if the authors had considered doing this not just to trigger reactions but to change pH etc… it could be very interesting to generate a pH range across the grid and see how the protein responds.

We thank the reviewer for this valuable suggestion. We are currently exploring experiments across different pH ranges and other variable parameters. However, the main objective of the present study is to demonstrate the feasibility of the approach and to establish a framework for its application to biologically relevant systems. A comprehensive investigation of these parameters for a specific biological question is beyond the scope of this manuscript and will be pursued in future studies.

I was interested in how the particles themselves maybe of better quality by this approach. There is some evidence (mainly discussed at conferences) that by using these different approaches the quality of the data itself can be improved. This is hard to quantify but trends can be seen with lees particles removed for grids made on the chameleon for example.
I noticed for TMV that a lot of particles went into the final reconstruction. For the other datasets after the initial 2D run where poorly picked particles and "junk" are removed what is the % of particles that make it through to the end and how does this compare to datasets published using the vitrobot for example?

We thank the reviewer for their comments. Supplementary Table 1 provides a quantitative comparison of particle numbers, retention ratios, and final reconstruction resolutions for multiple specimens (TMV, apoferritin, TRPM4, and streptavidin) prepared using cryoWriter and conventional plunge freezing. Particle retention varies across samples and experimental conditions, reflecting specimen-specific behavior and writing parameters, but remains sufficient to support reliable 3D reconstructions across all tested systems. For TMV, we achieved a reconstruction at 2.08 Å using only 25,681 segments, corresponding to a retention rate of 5.3% (Suppl. Table 1). This demonstrates that even with a relatively small number of particles, high-resolution reconstructions can still be obtained, comparable to those achieved using other freezing approaches.

No conflict of interest is stated but are any of the authors are also involved with the commercialisation of the cryoWriter and would this form a conflict?

Upon submission of the first version of this manuscript, none of the authors had a conflict of interest. In the meantime, HS has joined the board of the cryoWrite AG. The Competing Interests statement has therefore been extended with: "HS has joined the board of the cryoWrite AG in Feb. 2026, after submission of the initial version of this manuscript. The other authors declare no competing interests."

Minor edits
There are some cases of one or two sentence paragraphs such as the third paragraph of the results it feels like these could possibly be incorporated in to other paragraphs.

We thank the reviewers for noticing the errors. Now we have modified the manuscript accordingly.

H. sapiens and E. coli should be in italics.

We thank the reviewer for pointing out the errors. We have changed it to italics in lines 447, 476, 478, and 479.

Occasionally it states cryoEM rather than cryo-EM

We agree, we have changed it to cryo-EM in line 470

Figure S7, for the FSCwhy does this extend to 1.3A, it would be more appropriate to extend to ~3A.

We agree, the FSC plot now extends to 3 A. We changed the box size to reduce the x-axis to ~3A. The final reconstruction now gives us 4.97 A

Reviewer #2 (Remarks to the Author):

The paper "CryoWriter: A Robotic Solution for Improved Cryo-EM Grid Preparation" by K.V. Chinmaya describes their experience of using a new instrument for cryo-EM grid preparation and its potential application for time-resolved cryo-EM studies. The authors characterized how the parameters of the sample preparation technique affect the properties of the amorphous ice and also demonstrated high-resolution structures of several biomolecules. The work provides a well-structured study that will be interesting for a broad audience as a single instruments provides an opportunity to both prepare high quality samples and perform time-resolved studies. However, it would be more convincing if several intermediates at different times were shown to demonstrate real time-resolved possibility.

We thank the reviewer for the kind positive comment.

Comments:

1. When the authors apply 2 samples at the same grid and wait 200 ms before freezing, did they observe a set of intermediate structures that correspond to different times after the interaction?

No, we did not observe intermediate structures. The 200 ms delay is referring to the fastest speed, at which the cryoWriter can plunge-freeze the grid after deposition of the sample on the grid. The time to move the grid from the launchpad to the ethane cup for freezing is estimated to be 200 ms.
We designed this experiment to demonstrate on-grid mixing of a protein–ligand interaction and to assess whether binding between streptavidin and

biotin could be detected directly on the grid. We used a biotin concentration of 3 mM, which is a relatively high concentration, to rapidly initiate binding kinetics with streptavidin. In addition, we collected a relatively small dataset (2,209 micrographs) and used only 47,564 particles for the final 3D reconstruction. We are currently extending this approach to time-resolved experiments with broader biological applications. This study, therefore, serves as a proof of concept, demonstrating that time resolutions in the range of approximately 200 ms to 5 s can be achieved using this method.

2. In the movie that demonstrated the U-shaped movement of the capillary, the liquid with the first sample distributes quite a lot, and when the capillary distributes the second sample, it is seen that it mixes with the first sample. Why didn't the authors observe a mixture of both biomolecules everywhere?

We used self-wicking grids to observe on-grid mixing. The sample distribution on the self-wicking grids is controlled by glow-discharge time, writing speed, and writing volume. When the first sample is written on the grid, the sample spreads faster than the second since the grid saturates the amount of liquid. After the second writing, the capillary ends with a flag as seen in the video, where users will know the end of writing. The first two and last two squares do not mix. For this experiment, we decreased the glow-discharge time to reduce the liquid more slowly in order to see NrS-1 and apoF alone in the first and last grid squares and mixing regions in the middle.

The SI Video4 demonstrates the overlay of the atlas from the electron microscope and the video writing of two samples for NrS-1 and apoF mixing.

3. The authors showed that the ice thickness depends on many parameters, such as the capillary movement speed; time after applying the sample, temperatures, and so on. Did the authors consider selecting the parameters such that they would have a uniformly thin amorphous ice layer, which could be beneficial for imaging small molecules?

After finding parameters working consistently for us, we used these for the bulk of the experiments to ensure comparability. The current protocol allows for consistent preparation of grids with ice thicknesses as shown in the manuscript. We choose to use these parameters since they result in ice gradients giving us thicker and thinner areas, which, based on the sample applied, can be chosen accordingly. In our experience, 5-10 squares with the right ice thickness allow for the acquisition of millions of particles.

Minor comments:

1. Sometimes the same information is repeated in neighboring paragraphs, such as about the temperature of the system (pages 4 and 5) and about the increasing concentration of salts that is causing denaturation of the samples (pages 6 and 7).

We thank the reviewers for pointing out the repetition of the information in neighboring paragraphs. We changed the manuscript accordingly and removed the repeated information.

2. How accurately can the authors control the temperature of all the components of the system? If they were keeping the temperature of the grid 0.1 degree above the dew point, they could probably significantly reduce the degree of evaporation.

The temperature of the components can be set with 0.1 degree accuracy in the control software. We tested the actual temperature, and found this setting to be accurate within the accuracy of our equipment, however our testing equipment has an accuracy of 0.2 degrees.
For most of our experiments, the accuracy of the given temperature control was sufficient to either achieve almost no evaporation or a certain amount of evaporation which was desired, as described.

3. Did the authors estimate the degree of evaporation?

We performed a handful of experiments to estimate the evaporation rate. For a temperature offset of ~1° above the dew point temperature, which we commonly use, no evaporation was observable. For a 2° offset, we measured an evaporation rate of 1nl/s. For measuring, we write a spiral on the grid, and measure how long it takes for the liquid to completely evaporate.

4. It could be useful to indicate the actual temperature of the liquid ethane thermostat instead of indicating that it is slightly above -183C.

We keep the temperature between -180°C and -183°C, and the corresponding part in the manuscript was adapted accordingly.

5. The authors compare their instrument with different instruments for automatic cryo-EM grid preparation. However, it would probably be useful for the readers to include several sentences about existing methods for time-resolved cryo-EM to understand where the proposed technique stands (for example, a review of different methods is given in doi.org/10.1016/j.str.2022.11.014).

We added a comparison and contextualized our approach in relation to the current state of the art in this field in line number 328-336

REVIEWERS' COMMENTS

Reviewer #1 (Remarks to the Author):

I feel this will make for a valuable contribution to the field and the amendments made have improved the clarity and I feel it is suitable for publication.

We thank the reviewer for the positive comments.

Reviewer #2 (Remarks to the Author):

The authors of the paper "CryoWriter: A Robotic Solution for Improved Cryo-EM Grid Preparation" have significantly improved their manuscript and addressed most of the questions, however, a single questions remained:

1. "The SI Video4 demonstrates the overlay of the atlas from the electron microscope and the video writing of two samples for NrS-1 and apoF mixing." Unfortunately, The SI Video4 is not accessible in the "Manuscript Items" (please see the screenshot below):

We thank the reviewer for the positive comments. We have included two sample writings of NrS-1 and apoF mixing and an annotated video in **Suppl. Video 3.**
